# The intersection of nitrogen nutrition and water use in plants: new paths toward improved crop productivity

**DOI:** 10.1093/jxb/eraa049

**Published:** 2020-02-06

**Authors:** Darren C Plett, Kosala Ranathunge, Vanessa J Melino, Noriyuki Kuya, Yusaku Uga, Herbert J Kronzucker

**Affiliations:** 1 School of Agriculture and Food, The University of Melbourne, Melbourne, VIC, Australia; 2 School of Biological Sciences, University of Western Australia, Crawley, Perth, Australia; 3 Institute of Crop Science, National Agriculture and Food Research Organization, Tsukuba, Ibaraki, Japan; 4 Faculty of Land and Food Systems, University of British Columbia, Vancouver, British Columbia, Canada; 5 Nanjing Agricultural University, China

**Keywords:** Ammonium, aquaporins, *DRO1*, nitrate, nitrogen transport, phenotyping, root architecture, root barriers, suberin, water transport

## Abstract

Water and nitrogen availability limit crop productivity globally more than most other environmental factors. Plant availability of macronutrients such as nitrate is, to a large extent, regulated by the amount of water available in the soil, and, during drought episodes, crops can become simultaneously water and nitrogen limited. In this review, we explore the intricate relationship between water and nitrogen transport in plants, from transpiration-driven mass flow in the soil to uptake by roots via membrane transporters and channels and transport to aerial organs. We discuss the roles of root architecture and of suberized hydrophobic root barriers governing apoplastic water and nitrogen movement into the vascular system. We also highlight the need to identify the signalling cascades regulating water and nitrogen transport, as well as the need for targeted physiological analyses of plant traits influencing water and nitrogen uptake. We further advocate for incorporation of new phenotyping technologies, breeding strategies, and agronomic practices to improve crop yield in water- and nitrogen-limited production systems.

## Teaser

Given the critical importance and interconnectedness of water and nitrogen in determining crop yield, there is great impetus to understand and optimize their uptake. We review this intersection and provide proposals for improving these critical crop traits.

## Introduction

The two resources with the greatest influence on crop productivity are water and nitrogen (N). Food security is becoming more difficult to realize each year as the global population continues to increase, a problem compounded by the loss of arable land to urban sprawl, land degradation, and environmental limitations to agricultural production. Climate change has already had measurable impacts, increasing global temperatures and creating changing weather patterns. The impacts of these changes are only beginning to be understood, but it is clear that many agricultural regions will be impacted by increasingly hotter and drier conditions and more frequent weather extremes (IPCC, 2015). More than two-thirds of freshwater withdrawals and some 90% of total water consumption through human use globally are attributable to irrigation of crops, and water tables have been dropping at alarming rates in many parts of the world due to such water withdrawals (Britto and Kronzucker, 2018). Increasing global scarcity of water will also impact the way in which N fertilizer is accessed by plants and profoundly compromise crop productivity (Swarbreck *et al.*, 2019).

Water is critical to all stages of crop development. Upon sowing, seeds will not germinate without the presence of water. In dryland agriculture, sowing may occur into completely dry soil, but this is a strategy developed by farmers to ensure the seed is ready for germination upon the first rains. Quick emergence of seedlings from the soil is essential to maximize the capture of light, and roots must develop to secure water for this to occur. Plants in the vegetative growth stage are typically characterized by exponential growth, and this requires a large amount of water to sustain. Cell turgor, ion transport, enzyme function, and many other critical physiological roles within the plant are simply not possible without the presence of water. As water is a limiting resource in many regions of the world, the improvement of water-use efficiency (WUE) by crops is critical to maintaining food security. However, WUE is an extremely complex trait and has been relatively recalcitrant to manipulation efforts (Hatfield and Dold, 2019). It is clear that improvement of WUE in crops must be accompanied by agronomic strategies to reduce water requirements in agriculture.

Vast quantities of N fertilizer are applied by farmers every year to crops to maximize growth and yield. Among all nutrients, nitrogen is present in the largest quantities in plant tissue and is crucial to the development of plant structure, nucleotides, and enzymes, among many other central roles. However, crop plants are inherently poor at accessing applied N, taking up only 40–50% of the amount applied, or less (Raun and Johnson, 1999; Kronzucker *et al.*, 2000; Coskun *et al.,* 2017*a*, *b*). A large portion of the remaining amount can be lost to the environment in the form of volatilization of gaseous N compounds, such as ammonia and nitrous oxides, or washed out and leached to waterways, in particular in the form of nitrate, producing a substantial pollution load (Wang *et al.*, 2019). The production of N fertilizer through the Haber–Bosch process is also extremely energy demanding, as the process consumes an estimated 2% of the world’s energy supply (Erisman *et al.*, 2008; Pfromm, 2017). As a result, N fertilizer is expensive to produce and is the second greatest expense for farmers, next to fuel, resulting in large losses of potential income. Thus, it is clear that N-use efficiency (NUE) or crops must be improved for sustainable agricultural production; however, as for WUE, efforts to improve NUE have been stymied by the complexity of the trait in plants. Agronomic improvements in both traits are clearly necessary.

Several factors render improvements in WUE and NUE in crop plants challenging, and these include the multigenic nature of the traits, the need for phenotyping of traits throughout the crop’s life cycle, above- and below-ground, and under changing environments, and the fact that both traits are heavily influenced by interactions with environmental conditions and agronomic management practices (the so-called G×E×M interactions). As such, efforts to discover the genetic architecture of WUE and NUE in the field require the use of multiple field sites across a range of agro-climatic zones, over several seasons, to gain any statistical confidence in experimental outcomes. Compounding these practical difficulties is the lack of accurate phenotyping methods to measure complex traits in a meaningful manner. Both WUE and NUE can be broken down into a series of subtraits which can be measured with some accuracy. However, it is uncertain whether selecting varieties based on these subtraits will be useful in identifying improved WUE and NUE (Zubaidi *et al.*, 1999; Sadras, 2004, 2005; Sadras and Rodriguez, 2010; Garnett and Rebetzke, 2013; Asplund *et al.*, 2014; Sadras and Richards, 2014). Compounding this difficulty is that WUE and NUE are highly interdependent traits, which is not surprising, given that N compounds move through the soil as solutes. We will not review N or water transport individually, given the large number of reviews available on these topics (Britto and Kronzucker, 2002; Chaumont and Tyerman, 2014; Maurel *et al.*, 2015; Plett *et al.*, 2018).

In this review, we explore the role of water and N in determining crop yield, particularly where these roles intersect. Given their critical importance to plant growth and productivity, it can be difficult to disentangle the role of each individually, but this makes it all the more necessary to understand the influence of the interaction. We then offer suggestions for methods to improve water and N uptake in crops, as it is clear that both goals much be achieved on the road to improved crop yield.

## The intricate interaction between nitrogen uptake and water transport

### Mobility of nitrogen in soil by transpiration-driven mass flow and simple diffusion

While most N is taken up by higher plants from the soil as nitrate (NO_3_^−^) or ammonium (NH_4_^+^), most plants prefer NO_3_^−^ as long as the pH in the rooting zone remains favourable (Kronzucker *et al.*, 1997; Britto *et al.*, 2001; Britto and Kronzucker, 2002). The mobility of NO_3_^−^ ions in the soil is mainly governed by electrostatic interactions between negatively charged NO_3_^−^ ions and either soil minerals or soil organic matter (Allred *et al.*, 2007). The adsorption of anions to soil particles occurs when NO_3_^−^ ions become attached to positively charged exchange sites of soil. A significant percentage of exchange sites on soil mineral and organic matter are pH dependent. Under low-pH conditions, positively charged hydrogen ions (H^+^) become attached to certain exchange site functional groups, thereby causing these exchange sites to become positively charged (Foth, 1978; Bohn *et al.*, 2001). However, the mobility of abundant free NO_3_^–^ ions in soil depends on the water status or soil moisture content, which is a critical factor for the movement of not only NO_3_^–^ but also other mobile ions in the soil by both transpiration-driven mass flow and simple diffusion.

The extracellular matrix of the walls around living cells or the ‘apoplast’ is porous, the pores being filled with water in all but very exceptional circumstances (Münch, 1930; Steudle, 2000; Kim *et al.*, 2018). It is a physical continuum through which water and ions can freely move either by bulk/mass flow in the presence of a transpirational force, where ions, including NO_3_^–^ and NH_4_^+^, can be dragged by water (‘solvent drag’), or by simple diffusion in the absence of transpirational force. The tension created by the shoot during transpiration should propagate to the soil through roots due to a soil–plant–air continuum (SPAC). However, there is no evidence that mass flow plays a direct role in NO_3_^–^ uptake across the plasma membrane; the increased NO_3_ concentration in the rhizosphere due to solvent drag may enhance membrane NO_3_^–^ transport (Cernusak *et al.*, 2011; Matimati *et al.*, 2014). Thus, transpirational water fluxes appear to play a fundamental role in the acquisition of NO_3_^–^ and NH_4_^+^ as well as other mobile nutrients, explaining the functional up-regulation of membrane-embedded transporter proteins in plants grown in nutrient-deprived soils (Wilkinson *et al.*, 2007; Kupper *et al.*, 2012). Apparently, high transpirational water fluxes are primarily important for the acquisition of mobile nutrients or in zones where roots are sparsely distributed in the soil profile (Scholz *et al.*, 2007; Cramer *et al.*, 2008; Kupper *et al.*, 2012). Even though mathematical models have been used to predict and estimate the spatial extent of nutrient depletion around the rhizosphere (Rengel, 1993; Syring and Claassen, 1995), the magnitude of the distance over which mass/bulk flow is effective remains unknown. Knowledge of the spatial scale over which mass flow operates in soil is highly relevant to our understanding of plant nutrient acquisition from the soil, and there is a clear need for further research in the future.

### Ammonium in the paddy rice system

Nitrification and denitrification are the two major processes in determining the availability of soil N for crop plants during their growth and development (Cai, 2002; Kirk and Kronzucker, 2005). High-yielding lowland rice cultivars are often grown in waterlogged paddy fields. The rate of oxygen (O_2_) diffusion in waterlogged soil is 10 000 times lower than in air due to significantly lower diffusion coefficients (Nobel, 2009; Tuchscherr *et al.*, 2010). When O_2_ is depleted in the waterlogged soil, the remaining NO_3_^−^ is used by many microorganisms as a terminal electron acceptor, which results in the reduction of NO_3_^−^ to NH_4_^+^ (Tiedje *et al.*, 1982; Silver *et al.*, 2001).

Generally, the efficiency of N fertilizer uptake in lowland rice is poor, which is approximately as low as 20–40%, whereas upland crops often use 40–60% of the N applied to the soil (Vlek and Byrnes, 1986). Applied N fertilizer can also be lost by denitrification which is known to be one of the main pathways to lose N in flooded lowland rice fields (Reddy and Patrick, 1986; Chen *et al.*, 2013). Usually, ammonium-based fertilizers are the most common N fertilizers applied to lowland waterlogged rice fields (Chen *et al.*, 2013) and it is the commonly available N fertilizer for rice. Ammonium-based fertilizers such as urea can be biologically oxidized into NO_3_^–^ in the hypoxic top layers of the waterlogged soil due to nitrification; however, they undergo denitrification when NO_3_^–^ diffuses into anaerobic bulk soils (Tiedje *et al.*, 1982). This coupled nitrification and denitrification in flooded soils (i.e. rice paddies) has been demonstrated by many research groups (Reddy and Patrick, 1986; van Luijn *et al.*, 1996). Using mathematical calculations, it has been found that the diffusion of NH_4_^+^ and nitrification would minimize N loss from most flooded lowland soils, where NO_3_^−^ diffusion and denitrification usually occur at a rapid rate and are not likely to limit the overall process (Reddy and Patrick, 1986). The nitrification process in lowland rice is triggered by draining of floodwater, and accumulated NO_3_^–^ will be lost by alternate re-flooding and drainage (Reddy and Patrick, 1975; Cai, 2002). During rice fertilization, deep placement of NH_4_^+^-based N fertilizers into the anaerobic zones is recommended because it will reduce NH_4_^+^ nitrification thereby reducing N loss from the paddy soil (Bouldin, 1986), although spatial heterogeneity of N-enriched zones in rice paddy soils post-fertilization can be pronounced and must be considered in evaluating the effectiveness of fertilizer placements (Y. Li *et al.*, 2016). Although nitrification occurs in hypoxic zones of top layers and denitrification occurs in anaerobic zones of deep layers, NH_4_^+^ and NO_3_^−^ are usually co-present in lowland rice fields (Kronzucker *et al.*, 2000; Kirk and Kronzucker, 2005), and NO_3_^−^ can reach millimolar concentrations following fertilization (Arth *et al.*, 1998; Cai, 2002). However, the abundant and major form of inorganic N in lowland paddy fields is NH_4_^+^.

In addition to its abundance in paddy fields, NH_4_^+^ is the preferred N source over NO_3_^−^ for rice and many other plant species, which has often been attributed to the lower energy requirement for assimilation by roots (Bloom *et al.*, 1992; Balkos *et al.*, 2010; Ranathunge *et al.*, 2014). Rice also shows a superior tolerance to high NH_4_^+^ compared with other crop plants (Britto *et al.*, 2001; Britto and Kronzucker, 2002). However, NH_4_^+^ acquisition and translocation to the shoot can be enhanced by NO_3_^−^, and co-provision of the two N sources in the root zone can produce significant synergistic growth and yield effects (Kronzucker *et al.*, 1999).

### Effects of nitrate and ammonium supply on water transport/uptake of roots

Even 50 years after the pioneering research that discovered that water fluxes were important for nutrient uptake by mass/bulk flow and diffusion (Barber, 1962), the role of nutrients in regulating water fluxes in plants remains poorly understood (Raven, 2008). Several studies have suggested a possible role for xylem N concentration as a signal for the regulation of water fluxes in plants (Wilkinson *et al.*, 2007; Cramer *et al.*, 2009; Matimati *et al.*, 2014), but this idea lacks substantial experimental data support. Cramer *et al.* (2009) proposed a model of N regulation in which NO_3_^–^ modulates root hydraulic conductance through its control of plasma membrane-bound aquaporins, and foliar nitric oxide (NO) application modulates stomatal conductance (*g*_s_). These proposed models have emphasized the role of NO_3_^–^ in regulating water fluxes in plants (Wilkinson *et al.*, 2007; Kupper *et al.*, 2012) but neglected the potential regulatory effects of NH_4_^+^. However, given the importance of NH_4_^+^ fertilizers used in agriculture fields around the world, understanding of the regulatory effects of water fluxes by NH_4_^+^ fertilizers is critical and important.

Recently, significant progress has been made in understanding the regulation of hydrauli c conductivity of roots (Lpr) and plasma membrane-bound aquaporins under different NO_3_^−^ treatments in the model system Arabidopsis (G. Li *et al.*, 2016; Tyerman *et al.*, 2017). Comprehensive studies and investigations of the Lpr of Arabidopsis NO_3_^–^ transporter mutants including the transporters NRT1.1 (dual affinity) and NRT2.1 (HATS) showed that only the loss of *NRT2.1* reduced the Lpr. Even though Lpr was reduced in the *NRT2.1* knockout mutant, it still responded to low NO_3_^–^ supply. In the *NRT1.1* knockout mutant, there was a correlation between Lpr and shoot NO_3_^–^ concentration, but there was no apparent correlation between Lpr and root NO_3_^–^ concentration (Tyerman *et al.*, 2017). In the *NRT2.1* mutant, the transcript levels of *PIP1;1*, *PIP1;2*, *PIP2;1*, and *PIP2;3* showed a clear positive correlation between changes in Lpr and different NO_3_^–^ treatments (Tyerman *et al.*, 2017). Not only the transcript levels but also both PIP1 and PIP2 protein abundance were correlated with Lpr. Tyerman *et al.* (2017) also showed that root aquaporins which drive water flow through the transmembrane path were regulated by (i) shoot to root signal communication; (ii) shoot NO_3_^–^ status; and (iii) the function of the *NRT2.1* gene.

There is clear evidence that aquaporins play a role in the response to N, as indicated above. However, the change in transcriptional responses of root aquaporins to different external N status/concentrations is not instantaneous, but typically occurs over a period of several days (Tyerman *et al.*, 2017). Further, more caution is needed as transcription and protein amounts are not always correlated (Hachez *et al.*, 2012). For example, in tomato, altered Lpr to N changes in the medium were apparent within a very short time (Gorska *et al.*, 2008*a*, 2010), but aquaporin gene expression was not seen until 48 h after the treatment (Wang *et al.*, 2001). It is likely that Lpr changes in the early stages, due to modulation of water flow in the other pathways, namely symplastic through plasmodesmata and extracellular/apoplastic. In rice, switching from 10 ppm NH_4_^+^ to 0.5 ppm NO_3_^−^ resulted in a slight repression of *OsPIP1;1*, *OsPIP2;3*, *OsTIP1;1*, and *OsTIP2;2* expression. However, there was a major reduction in expression observed for *OsPIP2;4* and *OsPIP2;5*, whereas there was an induction for *OsTIP2;1* and *OsPIP2;6* (Tyerman *et al.*, 2017). In maize, addition of NO_3_^−^ was not reported to change *PIP* gene expression in roots within a 4 h time frame, while tungstate treatment, known as a potent inhibitor of nitrate reductase (de la Haba *et al.*, 1990; Britto and Kronzucker, 2005), greatly inhibited the expression of most *PIP* genes (Gorska *et al.*, 2008*b*). In Arabidopsis, a switch in exposure to NO_3_^−^ from NH_4_^+^ resulted in repression of *AtNIP2;1* gene expression, but the expression levels of all other aquaporins remained unchanged (Wang *et al.*, 2003). In another experiment, resupply of NO_3_^−^ to N-starved plants strongly induced a *TIP* member, and several others were induced weakly in Arabidopsis roots (Scheible *et al.*, 2004). These findings confirm that plant root water uptake can be altered by N but that this depends on N form, applied amount or concentration, and plant species. It remains unknown how these changes in membrane water permeability regulated by PIP aquaporins affect apoplastic and/or symplastic water flows. NRT1.2 seems to be an important candidate in the signalling of NO_3_^−^ to aquaporins, in a probably post-translational regulation mode affecting the activity of aquaporins modulating water flow through the plasma membrane. Physiological data suggest that potassium (K^+^) can directly reduce aquaporin-mediated N flow, while simultaneously improving plant WUE (Szczerba *et al.*, 2008; Balkos *et al.*, 2010; ten Hoopen *et al.*, 2010; Coskun *et al.*, 2013*b*; Britto *et al.*, 2014), offering another potential precedent of direct aquaporin regulation by a principal macronutrient ion. However, further comprehensive research is necessary to address whether (i) NO_3_^−^ and NH_4_^+^ affect the activity of PIP and TIP aquaporins in roots directly (e.g. by allosteric means); (ii) water flow through the apoplast and plasmodesmata responds to changing NO_3_^−^ and NH_4_^+^ levels; and (iii) signal transduction cascades are involved in aquaporin activity regulation in relation to shoot and root N levels.

### Formation of root apoplastic barriers made of suberin and lignin

Transport properties of roots are strongly related to their anatomy, and interpretation of water and ion transport measurement data requires detailed knowledge of root structure in order to understand function properly (Steudle and Peterson, 1998; Steudle, 2000; Ranathunge *et al.*, 2011*b*). In the past, and largely due to the difficulty of visualizing root structures *in situ* and accessing them without inflicting damage, scientists often left root structure/anatomy as ‘black boxes’. However, in the recent past, considerable progress has been made linking root transport properties with root structure. The ‘composite anatomical structure’ of roots results in ‘composite transport’ of both water and nutrient ions, including N (Steudle and Peterson, 1998; Ranathunge *et al.*, 2017). The parallel arrangement of the apoplastic (cell wall and extracellular) and symplastic (transmembrane and cell to cell) paths, and switches between these paths, are important features of this model (Steudle, 2000; Kim *et al.*, 2018). By switching between apoplastic and symplastic paths, depending on the prevailing resistances to water and nutrient flow, the composite transport model allows for an adjustment and for regulation of water and nutrient uptake driven by shoot demand. The apoplastic component of water and solute flow may be restricted by the existence of barriers such as Casparian bands. Along the cell to cell path (transmembrane and symplastic flow via plasmodesmata), aquaporins, ion transporters, plasmodesmata, and suberin lamellae all engage in the regulation of the intensity of water and solute flow (Tyerman *et al*., 1999, 2017; Roberts and Oparka, 2003; Ranathunge *et al.*, 2004).

Enhanced cell wall suberization and lignification are the most common and efficient strategies for sealing of roots under adverse conditions of both a biotic and abiotic nature. Suberization and lignification of roots are known to increase with age or developmental stage, and also during exposure to abiotic stresses (salinity, osmotic stress, drought, anoxia, heavy metals, nutrient stress, etc.) (Lux *et al.*, 2004; Kotula *et al.*, 2009; Krishnamurthy *et al*., 2009, 2011; Ranathunge *et al.*, 2011*a*; Kreszies *et al.*, 2019).

**Fig. 1.**
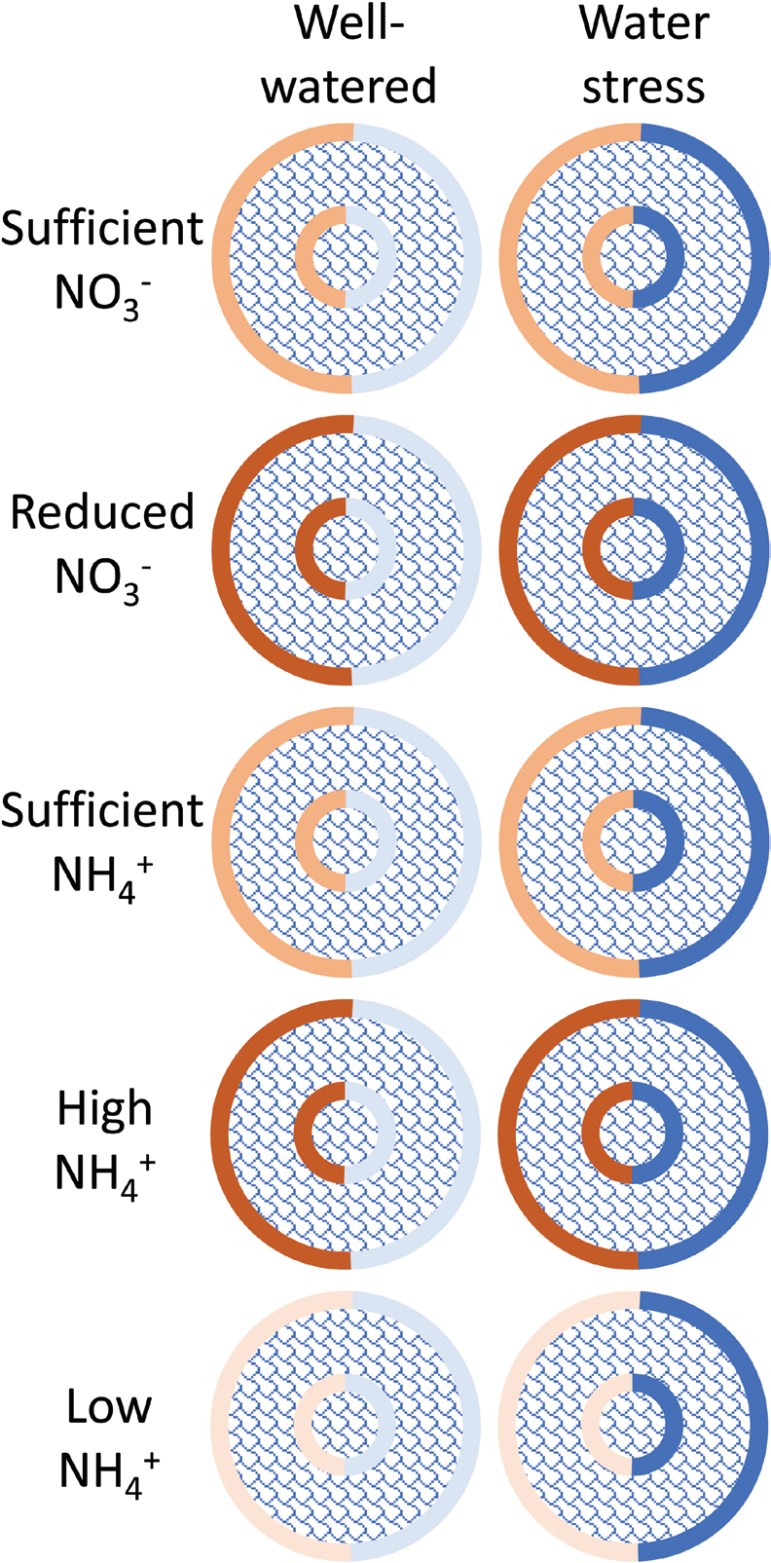
The putative impact of water and N supply on the development of plant root barriers. The outer and inner rings represent the exodermal and endodermal cell layers, respectively. The matrix represents the combinations of different water availability (columns) and different N sources and supplies (rows). Darker rings indicate induced root barriers which have increased suberin and lignin depositions in the cell walls, decreasing apoplastic water and N transport, while the reverse is depicted by lighter rings. The effect of N (left) and water (right) supply is represented on opposite sides of each ring. Future work must address whether an increase in barrier development influences N and water transport similarly, in particular where available water and N are having opposite influences on barrier development (e.g. low NH_4_^+^ combined with water stress, as depicted in the bottom right of the figure).

Our understanding of suberized and lignified apoplastic barrier deposition in roots, and the functions of these barriers, has made great progress in the past decade, but many gaps remain in our understanding of the metabolic and cellular processes of suberin and lignin formation. The relationship between suberin deposition and water transport is not always negatively correlated and this deserves further investigation to understand more completely (Kreszies *et al.*, 2018). Importantly, the impact of N supply on the formation of root barriers is not well understood (Schreiber *et al.*, 2005; Ranathunge *et al.*, 2016). In addition, no major studies have hitherto explored how combinations of stresses, such as N and water stress, will impact on the formation of root barriers. We have attempted to synthesize information about the putative combined effects of N and water on barrier formation in Fig. 1. Recently developed analytical methods, and the increasing availability of molecular tools for model systems in suberin and lignin research, will help fill these gaps in the future. Some variability in the characterization of barrier properties of suberized and lignified tissues indicates that the root barriers established in cell walls are complex and that there is no simple generalization to be made. A multifaceted approach is required, combining molecular genetics, analytical chemistry, and structural analysis with quantitative physiological transport studies to better understand the physiological importance of suberized and lignified cell walls in plants.

### C_3_ versus C_4_ plants—nitrogen and photosynthesis

Plants possessing the C_4_ photosynthetic machinery are able to concentrate CO_2_ around the Rubisco enzyme. This means that C_4_ species are able to reduce photorespiratory losses via this mechanism compared with C_3_ plants. The CO_2_-concentrating mechanism also allows for stomatal aperture to be reduced, resulting in less water loss via the transpiration stream, thereby increasing photosynthetic WUEs by 1.5–4 times over C_3_ species in similar conditions (Vogan and Sage, 2011). Related to this, photosynthetic N-use efficiency (PNUE) is generally 50–100% higher in C_4_ versus C_3_ plants (Sage *et al.*, 1987; Sage and Pearcy, 1987*a*, *b*; Ripley *et al.*, 2008; Vogan and Sage, 2011). However, in rice, a C_3_ species, there is evidence that N source can impact photosynthetic efficiency under water stress conditions. The provision of NH_4_^+^, as opposed to NO_3_^–^, under drought stress [simulated by polyethylene glycol (PEG)] allowed rice to maintain photosynthetic rate and Rubisco content (Guo *et al.*, 2007). However, in durum wheat, the capacity of N supply to increase photosynthetic parameters is heavily influenced by water supply, indicating the extent to which these two important determinants of crop productivity are linked (Shangguan *et al.*, 2000).

### Drought stress suppresses symbiotic nitrogen fixation

Legumes acquire N from symbiotic interactions with N_2_-fixing bacteria (rhizobia). The establishment of this symbiosis requires dual recognition and chemical communication that leads to the stimulation of nodule organogenesis (Oldroyd *et al.*, 2009). Once residing within the nodule, the infecting rhizobia can convert atmospheric N_2_ to ammonia. Grain legume species differ in their tolerance to drought stress, and the reader is directed to a number of excellent reviews (Subbarao *et al.*, 1995; Turner *et al.*, 2001; Turner, 2003). However, universally, the establishment of infection and symbiotic N_2_ fixation is very sensitive to drought stress (Sprent, 1971; Gil-Quintana *et al.*, 2013). A number of metabolic changes in the nodule of legumes exposed to water stress have been reported, including a decline in starch content and an increase in sucrose, decreased total free amino acid and ureide content (González *et al.*, 1995), proteolysis, and a decline in the content of the oxygen carrier leghaemoglobin (Guérin *et al.*, 1991), the latter causing poor oxygen diffusion in the nodule. It was suggested that ureides, which are N-rich compounds exported from the nodules of N_2_-fixing-tropical legumes (i.e. soybean and common bean), are involved in feedback regulation of the nitrogenase enzyme which is responsible for reduction of N_2_ to ammonia (Sinclair and Serraj, 1995; Serraj *et al.*, 1999). A more recent proteomic and metabolic approach suggests that this is unlikely (Gil-Quintana *et al.*, 2013). A more likely indirect regulation of nitrogenase via interaction between the ureide allantoin and abscisic acid (ABA) has been suggested. Allantoin was demonstrated to activate ABA production in Arabidopsis through increased transcription of *NCED3*, encoding an enzyme required for ABA biosynthesis and through post-translational activation of ABA (Watanabe *et al.*, 2014). The interaction between allantoin and ABA is also supported in legumes where exogenous supply of ABA to peas was shown to inhibit nodulation (Philips, 1971) and regulate nodule formation via suppression of Nod factor (a bacterial chemical signal released in the rhizosphere of a host plant), signal transduction, and cytokinin induction of the nodule primordia (Ding *et al.*, 2008). ABA likewise suppresses bacterial infection of the nodules (Ding *et al.*, 2008) and reduces N fixation rates by up to 80% (González *et al.*, 2001). The specific drought-induced suppression of nitrogenase activity via ureide-mediated induction of ABA is an area that requires further exploration.

### Nitrogen assimilation and remobilization under drought

Ammonium generated from metabolic processes such as RNA turnover (Zrenner *et al.*, 2006), protein turnover (Tabuchi *et al.*, 2007), and photorespiration (Mattsson *et al.*, 1997), as well as from uptake of external NH_4_^+^, is assimilated into organic N. Cytosolic NH_4_^+^ concentrations in the low to medium millimolar range are toxic to plants, although species differ in their sensitivity. Given that long-distance translocation of unassimilated NH_4_^+^ from the roots to the shoots rarely occurs, localized assimilation of NH_4_^+^ into organic N requires access to carbon skeletons, thereby inducing a localized carbon deprivation that contributes to the toxicity symptoms (Britto *et al.*, 2001; Britto and Kronzucker, 2002). Additionally, some plant species, such as barley, experience a high energetic burden associated with the futile cycling of NH_4_^+^, with NH_4_^+^ efflux constituting as much as 80% of primary influx (Britto *et al.*, 2001); such futile cycling, under special conditions, can also involve the NH_3_ species and passage through aquaporins (Coskun *et al.*, 2013*a*), pointing at another potential link to water fluxes.

Drought stress induces recycling of NH_4_^+^ via premature leaf senescence and enhanced photorespiration (Wingler *et al.*, 1999). The first step in recycling reduced N from NH_4_^+^ into organic molecules is catalysed by glutamine synthetase (GS). There are two isoforms of GS, cytosolic GS1 and plastidic GS2, both catalysing ATP-dependent condensation of NH_4_^+^ to the δ-carboxyl group of glutamate to form glutamine. GS has an important role in senescence-induced nutrient remobilization in cereal leaves (see reviews by Habash *et al.*, 2001 and Hirel *et al.*, 2007). Drought-afflicted rice plants have been reported to possess reduced total GS activity as the result of reduced transcript and protein levels of OsGS2, with the drought-tolerant rice cultivar Khitish better able to maintain total leaf GS activity than the drought-sensitive IR-64, over 12 d of water stress (Singh and Ghosh, 2013). It is understood that plants exposed to abiotic stresses such as drought undergo chloroplast dismantling into catabolic products such as amino acids and lipids, and further into nutrients that can be recycled and mobilized to sink organs (Otegui, 2018). It would be necessary to see if changes in GS2 protein levels, such as those reported by Singh and Ghosh (2013), are the result of plastidic protein turnover. Delaying chlorophyll degradation has been suggested to be a viable means of enhancing stress tolerance. Abiotic stresses induce expression of the chloroplast vesiculation pathway leading to chloroplast destabilization and the formation of vesicles. Silencing of the chloroplast vesiculation gene, *CV*, which interacts with the PSII subunit PsbO1, was shown to enhance drought tolerance of Arabidopsis (Wang and Blumwald, 2014) and rice (Sade *et al.*, 2018).

### ‘Haying-off’

**Fig. 2.**
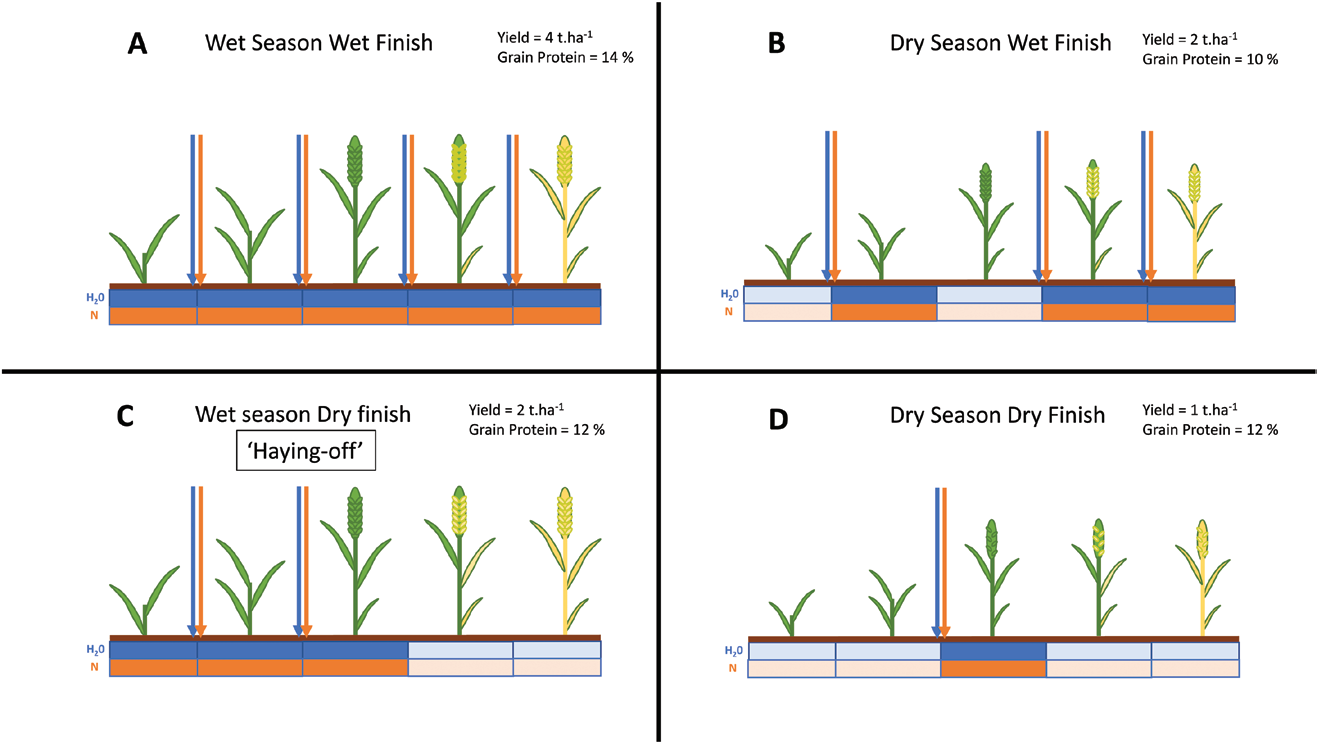
The importance of timing N application with water availability in dryland agriculture. Five stages of cereal crop development are represented in each panel. Arrows represent rainfall events (blue) and N applications (orange). Soil water (blue) and N (orange) for each development stage are represented by boxes beneath each developmental stage, indicating high water or N (dark), or reduced water or N (light). Size of plants and number of grains on each spike represent actual biomass and grain production of plants in each situation. Relative grain yields and protein content are provided for each of the four growth seasons. (A) A season with regular rainfall events; (B) a season with few rainfall events during vegetative growth, but regular rainfall during reproductive growth; (C) a season with regular rainfall events during vegetative growth, but few rainfall events during reproductive growth; (D) a ‘drought’ season with few rainfall events.

Rainfall in dryland agricultural regions is infrequent, and water shortages are common. Crops are generally produced in winter months when the majority of the rainfall events occur; however, this often results in crops maturing during months that are warmer and drier than those in which vegetative growth occurs. As a result, heat and drought stress are major factors during reproductive growth and often produce yield-limiting conditions. Of particular concern for farmers in these agro-climatic regions are production years with average or above-average rainfall during the vegetative growth stages which finish with drought conditions (Fig. 2). Crop plants produce vigorous vegetative growth and, as a result, use available soil water more quickly. Coupled with a dry finish to the season, this means crops are prone to ‘haying-off’, which refers to plants with a large biomass without accompanying large grain yield. The problem is compounded as the plants also produce grain with low quality. In the case of wheat crops, grain protein is critical to grain price; that is, low yields are exacerbated by reduced value per volume. Producers in such areas have adapted agronomic methods to deal with this type of growth environment. N fertilizers are applied in split applications over the course of the growing season, with limited amounts applied at sowing. This limits the vegetative growth in the early part of the season, and decisions to apply further applications are made based on rainfall events. If sufficient water will be available at the end of the season, producers will apply extra fertilizer to ensure the crops have nutrients available to maximize yield and grain quality. In drought years, these extra applications will not be applied to ensure some yield is achieved and the grain is of high quality. It should be noted that the converse situation can also occur in seasons with a wet finish, meaning the crops put on good yield with the extra water, but, if the producer has not applied sufficient N, the grain quality will have low protein content and the price per volume will be decreased as a result. (van Herwaarden *et al.*, 1998a, *b*; Garnett and Rebetzke, 2013)

### Molecular links

#### Water availability regulates nitrate transporters

The way in which N supply affects the N uptake system has been well characterized; in general the system is up-regulated by limiting N availability. It is also clear that, in the soil, N (particularly NO_3_^–^) supply is limited by water availability for movement of N towards the roots for uptake. The question of whether water limitation or the impact of drought on plants has an effect on the NO_3_^–^ transport systems has not been explored in depth. However, recent work suggests that mimicking the osmotic potential stress associated with drought by PEG treatment impacts the components of the NO_3_^–^ uptake system directly in rice. Expression levels of several *NRT2* genes were decreased, while the expression of the *NAR2* genes (*NRT3* genes) was increased by PEG treatment. Overexpression of *OsNAR2.1* had a positive impact on vegetative growth following treatment with PEG, and the transgenic lines had greater grain yield after drought treatment in a pot trial. Expression of genes associated with osmotic regulation in plants was altered by the overexpression of *OsNAR2.1*, suggesting that there are molecular links between the two regulatory systems (Chen *et al.*, 2019). Increased NH_4_^+^ supply improved the rate of water uptake and root hydraulic conductance, and increased transcript levels of several aquaporin genes in rice (Ishikawa-Sakurai *et al.*, 2014; Ren *et al.*, 2015). Further work suggests the signalling for these responses is ABA related (Ding *et al.*, 2016), as is the case for NO_3_^–^ (see below ‘Nitrate–ABA crosstalk’). It should be noted that urea and NH_4_^+^ (or ammonia) can be transported by certain aquaporins, and this represents a direct link between water and N transport (Liu *et al.*, 2003; Coskun *et al.*, 2013*a*; Kirscht *et al.*, 2016), and, while no plant example of NO_3_^–^-permeable aquaporins have been found, they do exist in mammals (Yasui *et al.*, 1999). Finally, NRT1.1 is expressed in root tips, but it is also expressed in guard cells, indicating a putative link between N supply and water transport in Arabidopsis (Guo *et al.*, 2003).

#### Recycling nitrogen under drought

The catabolism of nucleic acids and purine nucleotides in particular serves a housekeeping function, to recycle and remobilize nutrients during senescence (Taylor *et al.*, 1993; Hillwig *et al.*, 2011), thereby supporting plant growth and development (Zrenner *et al.*, 2006). Oxidation of the purine catabolite xanthine to glyoxylate liberates three molecules of CO_2_ and four molecules of NH_4_^+^. Purine catabolism via turnover of RNA is also induced under nutrient depletion stress (Taylor *et al.*, 1993; Melino *et al.*, 2018; Casartelli *et al*., 2019). Nitrogen-starved wheat plants exogenously supplied with the purine catabolites xanthine and allantoin grew and photosynthesized as well as plants re-supplied with NO_3_^–^, suggesting that they can support plant growth (Melino *et al.*, 2018).

**Fig. 3.**
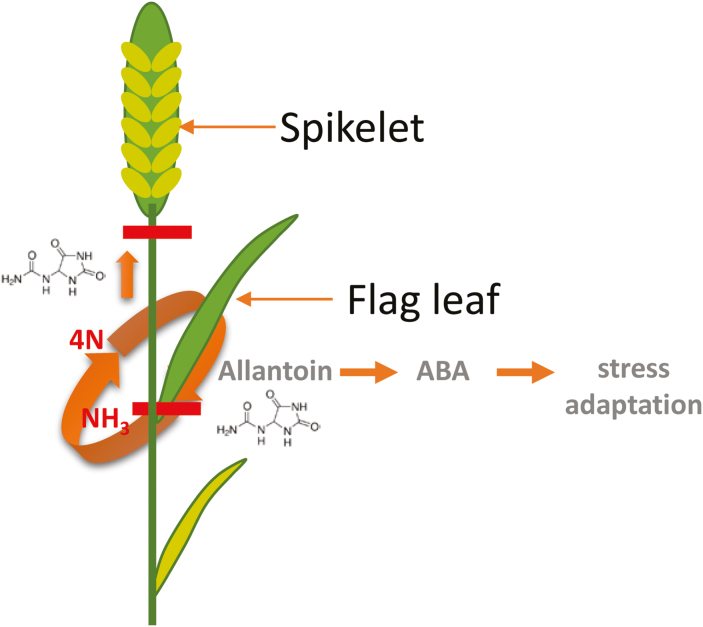
A plant metabolic link between adaption to drought and reduced N stress. Allantoin catabolism is restricted in drought-stressed plants. Allantoin accumulation both induces *de novo* synthesis of ABA and activates ABA from the inactive glycosylated form. Regulation of these processes may prevent loss of N as NH_3_ gas when carbon skeletons are in short supply. In contrast, under low N conditions, allantoin is catabolized (Melino *et al.*, 2018) and recycled to NH_3_ which can be reduced by N-assimilatory enzymes or instead serve as a cheap N storage form for translocation to the grain where it represents a significant portion of the soluble N pool (Casartelli *et al.*, 2019).

A correlation between the accumulation of ureide compounds, allantoin and allantoate, in response to drought stress has been demonstrated in a number of legumes, including common bean (Coleto *et al.*, 2014), soybean (Silvente *et al.*, 2012), and French bean (Alamillo *et al.* 2010), although this appears to be a response of only drought-sensitive genotypes (Coleto *et al.*, 2014). In comparison, allantoin accumulates in drought-tolerant cultivars of rice (Wang *et al.*, 2012; Degenkolbe *et al.*, 2013; Casartelli *et al.*, 2018) and wheat (Bowne *et al.*, 2012; Casartelli *et al.*, 2019). In fact, allantoin accumulation is understood to provide protection to non-leguminous plant species via induction of ABA as previously described (Watanabe *et al.*, 2014). This apparent contrast between the response of the ureide metabolic pathway in leguminous and non-leguminous plant species in response to water stress is unrelated to the *de novo* synthesis of ureides in nodules evidenced by the fact that ureides accumulate to levels in non-nodulated, NO_3_^–^-fed plants similar to those grown in symbiotic N-fixing conditions (Alamillo *et al.*, 2010). The accumulation of allantoin under drought in non-leguminous plant species, when carbon skeletons are limited for assimilation into organic N, has been suggested to prevent loss of that N as ammonia gas (Casartelli *et al.*, 2019) (Fig. 3).

#### Nitrate–ABA crosstalk

Soil moisture content can be heterogenous in distribution, and particularly so when partial root drying techniques are used to restrict crop water use. Movement of ABA from the roots, sensing water availability, to the leaves is an important coordinator of plant response to the environment (Dodd *et al.*, 2008). Likewise, soil N is often distributed heterogeneously in the soil, so that, when roots are grown under low-NO_3_^–^ conditions (e.g. 0.01 mM NO_3_^–^), lateral root branching is stimulated, particularly from the roots in direct contact with the NO_3_^–^ supply (1 mM NO_3_^–^) (Hackett, 1972; Drew *et al.*, 1973; Forde, 2014), although the response is genotype dependent (Liao *et al.*, 2006; Melino *et al.*, 2015). High concentrations of external NO_3_^–^ (>10 mM) inhibit lateral root development across the whole root, and this involves ABA signalling. Genetic dissection of the role of ABA in mediating the inhibitory effects of high NO_3_^–^ on root branching in Arabidopsis demonstrated the requirement for an ABA signal transduction pathway involving pathway genes *ABI4* and *ABI5* (Signora *et al.*, 2001). Signals such as dehydration stress (Xu *et al.*, 2012) or NO_3_^–^ (Ondzighi-Assoume *et al.*, 2016) also stimulate the release of bioactive ABA via β-glucosidase (BG1 or BG2). Conjugated forms of ABA (ABA-glucose ester, ABA-GE) are stored in the vacuole and transported in the xylem, and the active form of ABA must be released from the inactive conjugated state. Exposing Arabidopsis roots to an increased concentration of NO_3_^–^ (from 20 mM to 30 mM) led to a 3-fold increased ABA signal, with most of the accumulation localized to the endodermis and the stele of the growing tip as determined visually using a novel immunocytochemistry technique (Ondzighi-Assoume *et al.*, 2016). Genetic dissection of the NO_3_^–^-stimulated ABA accumulation demonstrated that it occurred even in the absence of *de novo* ABA biosynthesis and was dependent on an active BG1 which stimulated the release of ABA from ABA-GE. Nitrate was shown to regulate *BG1* at the transcriptional level (Ondzighi-Assoume *et al.*, 2016).

It has also been suggested that stress transduction pathways can regulate NO_3_^–^ sensing and signalling of the NO_3_^–^ transporters. Two members of the NO_3_^–^ transport peptide (NPF) family, *Medicago truncatula* NPF6.8 (Pellizzaro *et al.*, 2014) and Arabidopsis NPF4.6 (Kanno *et al.*, 2012), transport both NO_3_^–^ and ABA. Additionally, another member of this family, NPF6.3 (formerly NRT1.1 or CHL1), functions as an NO_3_^–^ sensor and transporter; the kinase CIPK23 and the calcium sensor CBL9 form a complex to phosphorylate NPF6.8 and activate NO_3_^–^ uptake at low external concentrations whilst dephosphorylation of NPF6.8 leads to a switch to low-affinity NO_3_^–^ uptake mode (Ho *et al.*, 2009). A protein phosphatase 2C family member, AtABI2, has been reported to be a positive regulator of AtNPF6.3 via interaction with and dephosphorylation of CIPK23 and CBL1. The ABA-insensitive Arabidopsis mutant, *abi2-2*, was shown to be defective in NO_3_^–^ perception (Léran *et al.*, 2015). It is interesting to consider that ABA, which is produced under drought stress, could restrict NO_3_^–^ sensing via ABI2 and NPF6.3 and thereby result in reduced NO_3_^–^ uptake (Léran *et al.*, 2015).

## Pathways to improving nitrogen and water uptake

### Better physiology

#### Uptake of water and nitrogen from the soil

It is clear that there is much left to understand about N movement in the soil and how this is influenced by the availability of water. Conversely, equally i mportant is the consideration of how soil water movement is regulated by plant nutritional status and the soil nutrient profile. Collaboration between root biologists and soil scientists will be required to truly unpack this interaction. The problem is complex given the connectivity and variation in soil types and characteristics, plant species, root ideotypes, crop physiology, environmental conditions, and agronomic management practices. It is clear that multi-level modelling will be useful in this regard, something which has only begun to be attempted in the context of the way plant water flux is regulated by nutritional factors (Cramer *et al.*, 2009).

#### Improved physiological techniques

A better understanding of the so-called subtraits making up WUE and NUE is required. A biomarker trait would be extremely useful in efforts to improve WUE and NUE in crops. To what extent the large phenotypes can be dissected into smaller and more easily measurable traits is unclear. In the case of NUE, we know that N uptake efficiency (NUpE) and N utilization efficiency (NUtE) (and remobilization efficiency in the case of grain protein crops) are important traits to improve, but to what extent improving GS activity, for example, will impact on NUE, is less understood. Complicating matters is that feedback inhibition of improved subtraits may make the improvements impossible to measure accurately in the manipulated plants. For example, measurement of integrative traits such as stable isotope discrimination for carbon and oxygen have shown some promise in their capacity to identify germplasms with superior WUE in field experiments (Cabrera-Bosquet *et al.*, 2011; Yousfi *et al.*, 2012), although the value of this relationship has shown variable results across studies (Condon *et al.*, 2004). Equally important is the identification of biomarkers that are actually useful in field-based experiments, as hydroponics and the use of PEG to mimic drought conditions can only be regarded as poor substitutes for the real-life interactions of water and N in soil.

#### Understanding the interaction of nitrogen and water uptake at the cellular level

Much remains to be explored regarding the interaction of N and water transport at the cellular level. For example, we need to understand the root zone and cell membrane localization of water and N transport more completely to determine if there is a common link with aquaporins, and new techniques such as single-cell transcriptomics, and measurement techniques to resolve co-location of transporters and physiological fluxes of water and N are required to answer these important questions. These types of integrated studies will allow identification of molecular identities of the basic machinery co-regulating water and N transport.

#### Improved WUE of crops through fine-tuning nitrogen supply

The understanding of the physiological, biochemical, and molecular mechanisms that control water use and WUE under N fertilization is critical for the development of new efficiencies of water use in agriculture. Even though grain yield and WUE in crops are primarily limited by the soil water deficit, higher yields are often achieved by using a higher dose of N fertilizer, especially in developing countries (Zhu and Chen, 2002; Wang *et al.*, 2018). However, this practice may result in negative environmental consequences. The understanding of the mechanisms that control water use and WUE under N fertilization is therefore not only critical for water-scarce areas, such as semi-arid and arid regions, but much more broadly.

Several studies have shown that N supply enhances plant productivity by improving WUE through: (i) reducing water loss by regulating stomatal conductance without impacting the assimilation rate (Toft *et al.*, 1989; Guehl *et al.*, 1995); (ii) increasing the assimilation rate as a result of increased N investment in the photosynthetic apparatus (Ranjith *et al.*, 1995) with no counterbalancing effect on stomatal conductance (Liu and Dickmann, 1996; Harvey and Van Den Driessche, 1999; Welander and Ottosson, 2000); (iii) causing a moderate increase in assimilation rate with a slight decrease in stomatal conductance (Wang *et al.*, 1998); or (iv) increasing root growth and root length density (RLD) in deeper soil layers (Zhang *et al.*, 2012). Further, several investigations showed that there is a positive correlation between NUE (van der Werf *et al.*, 1993) and drought tolerance in cereal crops. In winter wheat, lines with greater NUE showed higher drought tolerance ability under soil water deficit (Fan and Li, 2001). In maize, cultivars with either high NUpE and NUtE that linked to greater drought-tolerant ability produced consistently higher yields (Kamara *et al.*, 2014). In sweet sorghum, improved WUE and NUE under water stress contributed to the high degree of physiological acclimation to drought (Wang *et al.*, 2014). This finding highlights that higher NUE could help plants have a higher ability to tolerate drought stress.

In rice, high rates of N application to high-yielding rice increased WUE in conventionally flooded rice (Zhang *et al.*, 2012). Similarly, in wheat , application of N fertilizer significantly enhances root growth and RLD in deeper soil layers in wheat mainly because of (i) an increase of mineral N in deeper soil layers and (ii) a decrease in root mass per unit root length and average root diameter (Scott Russell, 1977; Liao *et al.*, 2004). N supply increased root growth and RLD in deeper layers of the soil profile, namely 80–140 cm, and improved water uptake, above-ground biomass, and WUE during the vegetative growth stage of wheat. Enhanced RLD in deeper layers of the soil profile proved to be a beneficial trait in environments that are prone to end-of-season drought or terminal drought because roots at deeper layers of the soil profile are able to extract available water from deep layers (Kirkegaard *et al.*, 2007; Palta *et al.*, 2011; Wang *et al.*, 2018). In wheat, the activity of these roots was evident as N applications enhanced water absorption of roots from deep soil layers more than in non-N supply treatments. In contrast, application of N fertilizer reduced the proportion of total biomass allocated to the root system, presumably because of the decreased average root diameter (RD) and root mass per unit of root length (RML) (Ercoli *et al.*, 2008; Kamiji *et al*., 2014). The resulting low RD and RML by higher N fertilizer application enhanced RLD without increasing root biomass; on the other hand, it significantly increased water uptake and above-ground biomass (Wang *et al.*, 2018). The proportion of the total biomass allocated to the root system was reduced by N fertilizer application compared with no N treatment. This lower partitioning of assimilates to roots has been positively correlated with higher grain yield and WUE (Fang *et al.*, 2010; Hu *et al.*, 2014).

#### Root traits that could improve water and nitrogen uptake

**Fig. 4.**
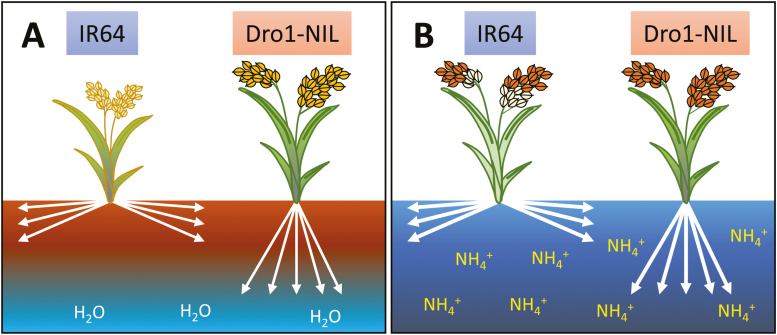
The beneficial impacts of deeper roots on water and N uptake in rice. Dro1-NIL has deeper rooting compared with the IR64 parental lowland cultivar. (A) The *DRO1* locus allows rice roots to explore deeper subsoil for water in a drought that IR64 cannot access, allowing Dro1-NIL to continue to grow and produce grain in drought seasons. (B) The deeper roots of Dro1-NIL allow the plants to access NH_4_^+^ in deeper subsoils, meaning the plants can access N later in the growing season to improve grain yield and quality as compared with IR64.

The genetic improvement of root traits could be valuable to enhance acquisition of water and nutrients because these resources are heterogeneously distributed in the soil (Hodge, 2004; Kitomi *et al.*, 2018; Y. Li *et al.*, 2016). In dryland agriculture, the water in the soil moves to the deep soil layers following gravity; that is, NO_3_^–^ dissolved in soil water is also leached by precipitation into deep soil layers. Therefore, deeper rooting represents an advantageous trait to capture water and N from subsoil (Trachsel *et al.*, 2013; Lynch and Wojciechowski, 2015). Under drought conditions, deep roots are especially advantageous in obtaining water efficiently from the subsoil (Rich and Watt, 2013). For example, a rice near-isogenic line (Dro1-NIL) which expresses deeper roots caused by a functional allele of *DRO1*, which is a quantitative trait locus (QTL) controlling root growth angle, had higher grain yield than the parent variety with shallow roots under drought condition (Uga *et al.*, 2013) (Fig. 4). In maize, the steep, cheap, and deep root ideotype consisting of specific architectural (reduced crown root number; longer, but fewer lateral roots) and anatomical (increased root cortical aerenchyma, altered root barriers) traits could also be useful to capture N efficiently from subsoil (Lynch, 2013; Lynch and Wojciechowski, 2015). Higher root length density also enhances N acquisition in some crops by increasing the root surface area (Garnett *et al.*, 2009). Whether the increased surface area contributes to N absorption depends largely on the soil environment; however, even in paddy fields where water and NH_4_^+^ are relatively equally distributed, increased deep roots in the lower soil layer enhanced grain yield in rice (Kawata *et al.*, 1978; Morita *et al*., 1986, 1988). The N uptake from the lower soil layer is important for grain filling in the maturity stage when N is often depleted in the upper soil layers (Toriyama, 2001). In fact, experiments using Dro1-NIL showed that deep rooting by *DRO1* improved N uptake after heading, resulting in better grain filling in a paddy field (Arai-Sanoh *et al.*, 2014). Root system architecture (RSA), which determines the extent of the root zone, has the greatest influence on a plant’s water and N acquisition area from the soil. However, efforts to improve crop uptake efficiency for water and N must consider the physiological function of roots as well as lateral roots and root hairs, and root anatomical traits. Since the interaction between roots, soil, and microorganisms is complex, there is no simple RSA ideotype for improving acquisition efficiency of water and N. To construct an RSA model adapted to each environment, it is necessary not only to characterize RSA of each crop and variety but also to understand soil conditions in the target environment. Wild accessions may be useful in this regard, but has been a relatively underexplored resource for improving root traits.

#### Understanding crosstalk between the two uptake systems

Our understanding of whole-plant molecular physiological links between water and N transport is rudimentary. Signalling cascades linking the potentiation of stress detection with action responses may occur across organs. For example, shoot-based signals indicating water or N deficiency must be transported to the roots to enhance uptake, therefore requiring an understanding and synthesis of the molecular events occurring across plant tissues and organs. Recent work identified EPIDERMAL PATTERNING FACTOR1 (OsEPF1) in rice, which regulates the stomatal patterning in leaves, but also controls the development of aerenchyma cells in roots (Mohammed *et al.*, 2019). This work represents an important link between regulation of gas exchange and potential drought adaptation with root transport physiology given the importance of root cortical development in the transport of nutrients, including N (Postma and Lynch, 2011; Hu *et al.*, 2014; Saengwilai *et al.*, 2014; Schneider *et al.*, 2017).

### Better phenotyping and breeding

#### Better phenotyping technology—both in the field and in controlled environments

High-throughput phenotyping technologies that can accurately measure physiological and morphological traits would be beneficial to efforts to improve the uptake efficiency of water and N. The choice between phenotyping in the field and controlled environments depends on the purpose and/or target traits. Since field conditions are heterogeneous, the data must be interpreted by taking into account the effects of the natural environment. Compounding this issue, phenotyping of large field trials requires a significant amount of resources in terms of labour, cost, and time. Nevertheless, field trials are indispensable for phenotypic selection in crop breeding. Recently, several types of field-based high-throughput phenotyping platforms have been established, from ground- to aerial-based platforms (Araus and Cairns, 2014; Shakoor *et al.*, 2017; Araus *et al.*, 2018). These platforms can acquire large amounts of phenotypic data non-destructively at one time with decreased labour and time costs compared with conventional methods. Ground-based platforms called ‘phenomobiles’ are vehicles with a range of on-board technologies such as navigation devices and sensors (Li *et al.*, 2019; Qiu *et al.*, 2019). The ground-based platforms are limited to measurement of a single or a few plots at a time. However, unmanned aerial platforms with multiple sensors, which can scan an entire trial in a short amount of time, have been developed (Zaman-Allah *et al.*, 2015; Santesteban *et al.*, 2017). This remote sensing technology based on visible/near-infrared spectroradiometry, infrared thermometry, and RGB colour cameras can acquire data on the physiological state of the plant body non-destructively, including water and N status, by different vegetation indices, such as the normalized difference vegetation index (NDVI) (Araus and Cairns, 2014; Kusnierek and Korsaeth, 2015).

Phenotyping of RSA in the field is more challenging because roots must be accessed from soil. Several sampling methods have been developed to date: the trench method for observation of vertical root distribution in the soil (Nemoto *et al.*, 1998; Uga *et al.*, 2013); methods of square or round monolith (Abe and Morita, 1994; Kano *et al.*, 2011); and soil-core methods for quantification of root parameters such as root volume or length for each soil depth. Other unique methods for quantifying root traits other than root volume and length include the basket method for measuring root growth angle (Uga *et al.*, 2011) and ‘shovelomics’ for scoring of several traits of basal roots after root sampling by shovels (Trachsel *et al.*, 2011). No method enables quantification of the entire RSA in the field at one time (Topp *et al.*, 2016). To do so, it is necessary to estimate the RSA by combining several methods (e.g. Nagel *et al.*, 2012; Guo and York, 2019). Development of high-throughput technology that can measure RSA non-destructively in the field will greatly facilitate efforts to improve traits such as nutrient capture.

In controlled environments, several types of high-throughput phenotyping platforms for above-ground traits have been established (Junker *et al.*, 2014; Halperin *et al.*, 2017; Czedik-Eysenberg *et al.*, 2018). Similar to the field, imaging data can be automatically acquired using several sensors fixed in a system installed in the greenhouse or growth chamber. These platforms enable non-destructive acquisition of data on plant traits including abiotic stresses such as water and N deficiency (Neilson *et al.*, 2015; Ge *et al.*, 2016). Various phenotyping methods or evaluating root traits related to RSA have also been developed for controlled environments using pot, box-pinboards, rhizotrons, and polyvinyl chloride (PVC) tubes, and hydroponic culture (Shashidhar *et al*., 2012; Downie *et al.*, 2015). These culture conditions generally provide a limited root zone, which can result in different root phenotypes compared with field-grown plants. Rice, which often grows in hypoxic conditions, is amenable to several high-throughput phenotyping systems for RSA that have been established based on 3-D imaging of roots developed in gel media or hydroponic growth systems (Iyer-Pascuzzi *et al.*, 2010; Clark *et al.*, 2011; Fang *et al.*, 2013; Uga *et al.*, 2018). Just as for root phenotyping in the field, however, these methods do not allow measurement of the whole picture of 3-D RSA in the soil. To address this problem, 3-D root image analysis using X-ray computed tomography (CT) and MRI have been developed (Metzner *et al.*, 2015; van Dusschoten *et al.*, 2016; Atkinson *et al.*, 2019). Phenotyping systems using X-ray CT and MRI imaging are still low throughput because their scanning and 3-D reconstruction require significant time for data acquisition and analysis. To use these methods practically, the speed and efficiency of their scanning and 3-D reconstruction require improvement.

#### Incorporation of multiple site–year trials into breeding efforts to improve WUE and NUE is required

Given the significant G×E×M interaction for both traits, it is unclear whether QTLs showing effects on improving the traits will be expressed or beneficial across different agro-climatic regions. As a result, models incorporating the G×E×M information may be useful in designing the genetic architecture of new varieties targeted to different environments. It is also clear, especially in dryland agricultural settings, that selecting for germplasms with superior NUE is not possible, and potentially pointless, unless the accompanying improvements in WUE are also selected. Efforts have been made to utilize existing networks for trials with proper characterization of climate and management variables and to model metadata (e.g. National Variety Trials in Australia; or the Wheat Genetic Improvement Network in the UK); however, there is room for improvement in this regard. Incorporation of new breeding targets may be required, for example breeding for growth habits such as altering maturity to take advantage of rainfall events. The complexity of the WUE–NUE interaction is emerging from efforts to understand the role of selection in the development of new varieties (Bänzinger *et al.*, 2000; Ribaut *et al.*, 2007; Sadras and Lawson, 2013; Elazab *et al.*, 2016; Prey *et al.*, 2018).

### Better agronomy

Farmers in dryland agricultural environments already manage drought by applying N in split applications only when there is sufficient soil water present to support vegetative or reproductive growth. These decisions are based on a coarse understanding of the effect of timing of N application on yield and require further study to evaluate the implications of various nutrient management decisions (Abid *et al.*, 2016). There is potential to prime the crop plants for various degrees of water availability based on the nutrient profile of fertilizer applied to induce useful modifications of root architecture. Similarly, in irrigated cropping environments, the capacity of crops to access fertilizer can be managed by managing irrigation decisions to maximize beneficial root architecture development (van Herwaarden *et al.*, 1998*a*, *b*; Garnett and Rebetzke, 2013). A similar optimization of both N and water acquisition may be achieved in flooded systems, such as irrigated rice fields, by imposing alternate wetting and drying protocols, which have been shown to shift soil microbial communities and optimize ratios of NH_4_^+^ and NO_3_^–^ in soil water by favouring nitrification during drying periods and inhibiting it during flooding periods while reducing water consumption (Kronzucker *et al.*, 1999; Kirk and Kronzucker, 2005). Novel precision agriculture approaches in these areas carry much promise.

Foliar application of fertilizer is much less dependent on the availability of water than soil application. Work has been carried out to evaluate the efficiency of foliar uptake compared with root uptake, the role of the type of N (NO_3_^–^, NH_4_^+^, urea) preferred by plants, and the composition of the application solution (e.g. use of adjuvants) versus soil application (Woolfolk *et al.*, 2002).

The potential impact of novel fertilizer technologies and soil amendments (e.g. silicon, ammonium chloride, biological nitrification inhibitors, novel slow-release fertilizer coatings or carriers, such as graphene) on NUpE is only beginning to be explored, but they have shown promise (Snyder, 2017). Technologies which time fertilizer release with water availability would have obvious benefit for crop growth and would reduce losses associated with N that has not been taken up by the crop.

## Conclusions and future work

Given the fundamental importance of water and N supply to the success of sustainable crop production and our ability to feed the world, it is daunting to realize how much is left to discover regarding the uptake of these resources by plants. The genetic regulation of water and N uptake individually is complex, but it is clear that efforts to improve N uptake must also take into consideration the intricate ties with water availability and uptake. It also must be acknowledged that climate change will alter agricultural systems in complex ways, and these changes must be understood as part of programmes to improve N and water transport in crops. While our understanding of the machinery of transport systems has grown significantly, the signalling pathways regulating the uptake of water and N are not yet sufficiently understood. Similarly, the responses of root barrier formation to water and N supply and how changes in both resources together affect this formation require further investigation. Despite the enormity of the task of improving WUE and NUE in crops, with well-designed physiological studies, improved phenotyping and breeding capacity, and development of cutting-edge agronomic solutions, we are confident substantial gains will be realized in the coming years.
